# Predictive factors of acute respiratory events during initial induction chemotherapy in patients with advanced neuroblastoma

**DOI:** 10.1002/cnr2.1499

**Published:** 2021-07-13

**Authors:** Motohiro Matsui, Atsushi Makimoto, Nobuhiro Nishio, Yoshiyuki Takahashi, Mitsuyoshi Urashima, Yuki Yuza

**Affiliations:** ^1^ Department of Hematology/Oncology Tokyo Metropolitan Children's Medical Center Tokyo Japan; ^2^ Division of Molecular Epidemiology Jikei University School of Medicine Tokyo Japan; ^3^ Department of Pediatrics Nagoya University Graduate School of Medicine Nagoya Japan

**Keywords:** acute respiratory distress syndrome, acute respiratory events, disseminated intravascular coagulation (DIC) score, lactate dehydrogenase (LDH), neuroblastoma

## Abstract

**Background:**

Acute respiratory events (ARE) occasionally occur during induction chemotherapy as a complication in patients with advanced neuroblastoma.

**Aims:**

The present study aimed to identify the predictive factors of ARE, defined as severe hypoxia, during initial induction chemotherapy in patients with newly diagnosed advanced neuroblastoma.

**Methods and Results:**

The medical records of 75 consecutive patients in whom stage III or IV neuroblastoma was newly diagnosed between January 2003 and December 2018 at two medical institutions were retrospectively reviewed. The outcome was ARE, which were assessed by measuring oxygen saturation between days 1 and 14 of initial induction chemotherapy. Severe hypoxia was defined as grade 3 or higher according to the Common Terminology Criteria for Adverse Events version 4 (CTCAE v4.0) or decreased oxygen saturation at rest (e.g., pulse oximeter <88% or PaO_2_ ≤55 mmHg). Possible predictive factors on admission were first screened for using univariate analyses with *P* = .05, then models of the predictive power of the outcome were evaluated by generating receiver operating characteristic (ROC) curves. Eleven patients (14.7%) had the outcome, including three (4.0%) who required respiratory support in the intensive care unit. The area under the curve of the ROC for the predictive factors screened by univariate analyses was 0.84 (95% confidence interval [CI]: 0.73–0.95) for lactate dehydrogenase (LDH) and 0.90 (95% CI: 0.82–0.98) for the disseminated intravascular coagulation (DIC) score.

**Conclusion:**

The LDH value and DIC score on admission may be clinically useful predictors of ARE during initial induction chemotherapy in patients with advanced neuroblastoma.

**Abbreviations**
ARDS: acute respiratory distress syndrome
ARE: acute respiratory events
AUC: area under the curve
DIC: disseminated intravascular coagulation
INSS: International Neuroblastoma Staging System
IQR: interquartile range
LDH: lactate dehydrogenase
NUH: Nagoya University Hospital
OR: odds ratio
PT: prothrombin time
ROC: receiver operating characteristic
TLS: tumor lysis syndrome
TMCMC: Tokyo Metropolitan Children's Medical Center

## 1 INTRODUCTION

Neuroblastoma is an aggressive childhood cancer with a poor prognosis in the advanced stages. Multidisciplinary treatment, including multiagent chemotherapy, surgery, radiotherapy, hematopoietic stem cell transplantation, and immunotherapy, aimed at minimizing residual disease, has increased the long‐term survival rate of patients with high‐risk neuroblastoma to approximately 50%.^1^, ^2^, ^3^ Among the variety of treatment modalities, multiagent chemotherapy consisting of vinca alkaloid, anthracyclines, alkylators, and platinum is still the mainstay.^4^ Accurate control of adverse events during the initial induction chemotherapy is necessary but difficult because the condition of the patients is frequently unstable due to the huge tumor burden from both the primary tumor and metastatic sites. For this reason, acute respiratory events (ARE) during induction therapy possibly stemming from tumor lysis are occasionally observed.

The incidence of acute respiratory distress syndrome (ARDS), which is considered a severe form of ARE occurring during induction chemotherapy for advanced neuroblastoma, is reportedly one in 86 (1.2%)^6^ to 1 in 46 (2.2%)^5^ based on previous case series. ARDS was not reported in stage 1 and 2 disease in several studies.^7^, ^8^, ^9^ The present, retrospective, cohort study conducted at two, high‐volume centers in Japan aimed to find the predictive factors of ARE to enable their prediction prior to induction chemotherapy.

## 2 PATIENTS AND METHODS

The present, retrospective, cohort study was performed using data from the medical records of patients who received the diagnosis of neuroblastoma between January 2003 and December 2018 at Tokyo Metropolitan Children's Medical Center (TMCMC) or Nagoya University Hospital (NUH).

Patients were enrolled if they had newly diagnosed, histologically‐proven, International Neuroblastoma Staging System (INSS) stage 3 or 4 neuroblastoma^10^ and no history of previous antitumor treatment. Intermediate risk and high risk were defined in accordance with the International Neuroblastoma Risk Group Classification System.^11^ MYCN amplification was determined by fluorescence in situ hybridization.^12^ Fever preceding induction therapy was defined as grade 1 or higher according to CTCAE v4.0 (e.g., a single temperature reading equal to or higher than 38.0°C) during the first week before induction therapy. The outcome was ARE, defined as severe hypoxia, including ARDS, between days 1 and 14 of the initial induction chemotherapy. Severe hypoxia was defined as grade 3 or higher according to CTCAE v4.0 or decreased oxygen saturation at rest (e.g., pulse oximeter <88% or PaO_2_ ≤55 mmHg). Because many of the values required for arterial blood gas analysis were missing, oxygen saturation alone was used to assess the respiratory disorders. The definition of the Pediatric Acute Lung Injury Consensus Conference^13^ was applied to confirm the diagnosis of pediatric ARDS. Patients with hypoxia caused by the comorbidities of pneumonia and bacteremia, which were respectively diagnosed by chest computed tomography and blood culture, were excluded. Clinical data, including laboratory data, such as the lactate dehydrogenase (LDH) value, were extracted from the electronic medical records. The Japanese Ministry of Health, Labour and Welfare's old disseminated intravascular coagulation (DIC) diagnostic criteria, which includes underlying diseases, clinical symptoms, platelet count, fibrin‐related markers, fibrinogen, and prothrombin time (PT) ratio, were used to derive the DIC score.^14^ All the data used were derived from hospitalized patients. Continuous variables were expressed as the median and interquartile range (IQR). Discrete variables were expressed as a frequency and percentage. Logistic regression analysis was used to screen for predictive factors of ARE. For each significant variable, an odds ratio (OR) with a corresponding 95% confidence interval (95% CI) and *P* value were computed. *P* < .05 was considered to indicate statistical significance. The discriminatory power of the model was assessed using the receiver‐operating characteristic (ROC) curve and the area under the curve (AUC). An AUC of 0.5 indicated no discrimination, 0.7–0.8 was considered acceptable, 0.8–0.9 was considered excellent, and more than 0.9 was considered outstanding. The sensitivity was set at the rather stringent level of above 90% because of the importance of preventing ARE. All the data were analyzed using Stata, version 16.0 (StataCorp LLC).

The present study was conducted in accordance with the Helsinki Declaration of the World Medical Association and Ethics Review Procedures concerning Research with Human Subjects. The protocol was approved by the Ethics Committee at TMCMC. The requirement for informed consent was waived because the data were anonymized and the study was retrospective. All the data were subject to a strict privacy protection policy with an opt‐out clause.

## 3 RESULTS

### 3.1 Study population

In total, 75 patients with newly diagnosed neuroblastoma who met the inclusion criteria during the study period were identified. Table 1 shows the characteristics of the patients at baseline. Thirty‐five (46.7%) patients were female, and the median age was 2.7 (IQR 1.3–3.8) years. Most patients had stage IV (85.3%) neuroblastoma and a high risk (86.7%).

**TABLE 1:** Characteristics of the 75 patients with newly diagnosed neuroblastoma

Age (years)	2.7 (IQR 1.3–3.8)
Sex, n (%)	
Female	35 (46.7)
Male	40 (53.3)
Institution, n (%)	
NUH	52 (69.3)
TMCMC	23 (30.7)
Stage, n (%)	
3	11 (14.7)
4	64 (85.3)
Risk, n (%)	
Intermediate	10 (13.3)
High	65 (86.7)
MYCN amplified, n (%)	
Yes	23 (30.7)
No	48 (64.0)
Unknown	4 (5.3)
Treatment, n (%)	
Regimen A^29^	50 (66.7)
COG A3961^8^	8 (10.7)
Rapid COJEC^30^	10 (13.3)
Others	7 (9.3)
Primary site, n (%)	
Adrenal gland	47 (62.7)
Retroperitoneum	21 (28.0)
Mediastinum	7 (9.3)

Abbreviations: NUH, Nagoya University Hospital; TMCMC, Tokyo Metropolitan Children's Medical Center; COG, Children's Oncology Group.

Forty (53.3%) patients were treated with regimen A consisting of cyclophosphamide, vincristine, pirarubicin, and cisplatin.^29^ Ten (13.3%) patients were treated with rapid cisplatin, vincristine, carboplatin, etoposide, and cyclophosphamide (COJEC).^30^

### 3.2 Characteristics of patients with acute respiratory events

ARE were observed in 11 (14.7%) of the 75 patients. Table 2 describes the patients' characteristics. Eight and three patients had grade 3 and grade 4 hypoxia, respectively. Three (#10, #56, #68) experienced ARDS and required respiratory support in the pediatric intensive care unit (PICU). One (#10) of the three patients received veno‐arterial extracorporeal membrane oxygenation. Two (#10, #56) had to discontinue chemotherapy temporarily, and all three patients needed to delay their second course of chemotherapy. None of the patients died during induction therapy.

**TABLE 2:** Characteristics of 11 patients with acute respiratory events

No.	Age (years) /gender	Stage	Primary tumor site	Induction protocol	Hypoxia grade	Severe hypoxia onset after induction (days)	Length of stay in ICU (days)	MYCN amp	DIC Score	LDH on adm (mg/dl)	CT findings
1	2.2/M	4	Adrenal gland	VCR + CPA	3	5		No	2	1359	NA
8	8.4/M	4	Adrenal gland	A regimen	3	1		NA	1	731	Pleural effusion
10	7.7/M	4	Adrenal gland	A regimen	4	5	12	Yes	5	2660	ARDS/ Pleural effusion
20	9.4/M	4	Adrenal gland	A regimen	3	1		Yes	NA	1910	Pleural effusion
24	1.4/M	4	Adrenal gland	A regimen	3	5		Yes	3	3116	Pericardial effusion
33	1.2/M	3	Retroperitoneum	A regimen	3	1	3	Yes	4	5706	Pleural effusion
48	3.5/M	4	Adrenal gland	A regimen	3	1		Yes	NA	1144	NA
50	1.6/M	4	Adrenal gland	A regimen	3	12		Yes	3	4581	Pericardial effusion
56	3.2/M	4	Adrenal gland	A regimen	3	4	8	Yes	6	3196	ARDS
68	0.8/M	4	Adrenal gland	A regimen	4	5	15	No	4	4830	ARDS
72	2.5/M	4	Adrenal gland	A regimen	4	1	17	Yes	1	4147	Pleural effusion

Abbreviations: ICU, Intensive Care Unit; DIC, disseminated intravascular coagulation; LDH, lactate dehydrogenase; CT, computed tomography; ARDS, acute respiratory distress syndrome; NA, not available.

Five of the 11 patients experienced ARE on the first day of induction therapy (i.e., early onset) while the remaining six patients experienced ARE on the fourth day or later (i.e., late onset). Pleural effusion occurred in four of the five patients with early onset and in one of the six patients with late onset. On the other hand, ARDS and pericardial effusion was observed in three and two of the patients with late onset, respectively, but in none of the patients with early onset.

### 3.3 Screening for predictive factors of acute respiratory events

The baseline characteristics of the patients with ARE were compared with those without ARE. Univariate analysis identified a high LDH value (*P* = .001) and high DIC score on admission (*P* = .006), fever preceding induction therapy (*P* = .012), and MYCN amplification (*P* = .003) as possible risk factors of ARE in patients with newly diagnosed neuroblastoma (Table 3). Uric acid, creatinine, and ferritin values and the primary tumor site were not associated with ARE (Table 3).

**TABLE 3:** Univariate analysis of risk factors of acute respiratory events (ARE) in patients with newly diagnosed neuroblastoma

	ARE (N = 11)	No ARE (N = 64)	*P* value
Age, years (IQR)	2.5 (1.4–7.7)	2.7 (1.3–3.7)	.816^a^
Female sex, n (%)	3 (27.3)	32 (50.0)	.174^b^
LDH on admission (IQR)	3116 (1359–4581)	702 (426–1607)	**.001** ^a^
Pre‐induction fever, n (%)	9 (81.8)	23 (35.9)	**.012** ^b^
CRP on admission (IQR)	4.4 (2.4–11.2)	2.0 (0.3–5.3)	.103^a^
Ferritin on admission (IQR), N = 42	261.5 (111–629), N = 6	204 (65.5–375.5), N = 36	.215^a^
Primary tumor site:			
Adrenal grand, n (%)	10 (90.9)	37 (57.8)	.065^b^
Retroperitoneum, n (%)	1 (9.1)	20 (31.3)	.162^b^
Mediastinum, n (%)	0 (0)	7 (100)	–
MYCN amplified, n (%), N = 71	8 (72.7), N = 10	15 (23.4), N = 61	**.003** ^b^
NSE on admission (IQR), N = 74	568 (290–721), N = 10	298 (116–430), N = 64	.418^a^
DIC score (IQR), N = 46	3 (2–4), N = 6	0 (0–1), N = 40	**.006** ^a^
Regimen A (%)	10 (90.9)	40 (62.5)	.065^b^
Liver metastasis, n (%)	2 (18.2)	8 (12.5)	.611^b^
Bone marrow metastasis, n (%)	5 (45.5)	39 (84.8)	.340^b^
Stage 4, n (%)	10 (90.9)	54 (84.4)	.577^b^
High risk, n (%)	11 (100)	54 (84.4)	.159^b^
Bone metastasis, n (%)	6 (54.5)	37 (57.8)	.840^b^

*Note*: **Bold type** indicates significant *P* value.

Abbreviations: ARE, acute respiratory event; LDH, lactate dehydrogenase; CRP, C‐reactive protein; NSE, neuron‐specific enolase; DIC, disseminated intravascular coagulation; JNBSG, Japan Neuroblastoma Study Group.

^a^ Mann–Whitney *U*‐test.

^b^ Chi‐square test.

### 3.4 Receiver operating characteristic curve of the lactate dehydrogenase and/or disseminated intravascular coagulation score for acute respiratory events

**FIGURE 1:**
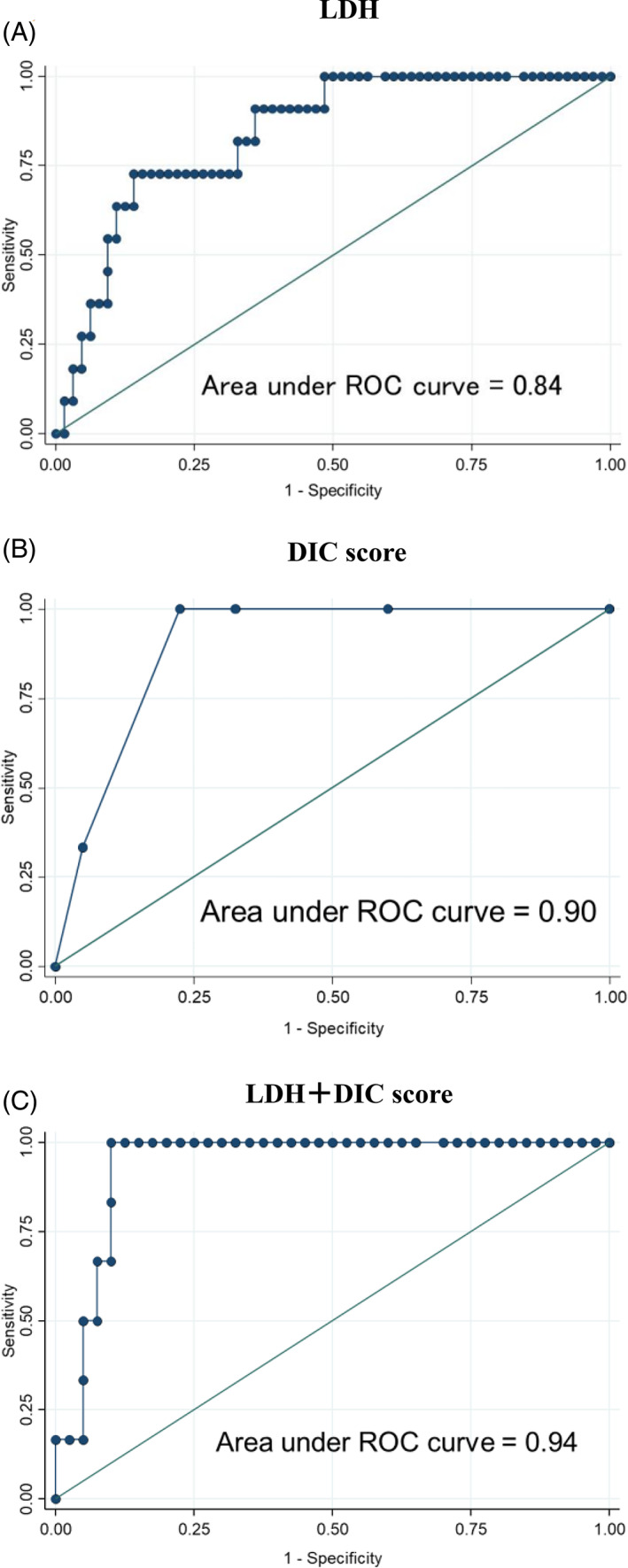
Receiver operating characteristics curves for neuroblastoma prediction. (A) using the LDH value (N = 75), (B) using the LDH value and DIC score (N = 46), (C) using the DIC score (N = 46). The formulae for calculating ARE risk using the two scores are as follows: ARE risk = exp(X)/(1 + exp[X]). (A) LDH scoring system X = LDH × 0.0006531–3.057099. (B) LDH and DIC scoring system X = LDH × 0.0004556 + DIC score×1.40751–6.70864. (C) DIC scoring system X = DIC score × 1.678482–6.048829

Because the LDH value, DIC score, fever preceding induction therapy, and MYCN amplification were found to be statistically significant continuous variables on univariate analysis, they were chosen as candidate predictive factors of ARE during induction therapy. The AUC of the LDH value was excellent at 0.84 (95% CI: 0.73–0.95; N = 75) (Figure 1(A)). The AUC of the DIC score was outstanding at 0.90 (95%CI: 0.82–0.98; N = 46) (Figure 1(B)). The AUC of fever preceding induction therapy and MYCN amplification were not good at 0.73 (95% CI: 0.60–0.86; N = 75) and 0.78 (95% CI: 0.64–0.92; N = 71), respectively. The optimal cutoff points at above 90% sensitivity were 1144 mg/dl for LDH and 3 for the DIC score with a sensitivity and a specificity of 90.9% and 64.1% and 100% and 77.5%, respectively (Figure 1(C)). The positive and negative predictive values were 30.3% and 97.6% and 40% and 100%, respectively (Table 4).

**TABLE 4:** Diagnostic accuracy of LDH and DIC for ARE

	LDH (1144)	DIC (≧3)	LDH and DIC
Sensitivity	90.9%	100%	100%
Specificity	64.1%	77.5%	90%
Positive predictive value	30.3%	40.0%	60%
Negative predictivevalue	97.6%	100%	100%

Abbreviations: ARE, acute respiratory event; LDH, lactate dehydrogenase; DIC, disseminated intravascular coagulation.

A binary logistic regression model was used to combine the LDH value and DIC score. This combination yielded an AUC of 0.94 (95%CI: 0.87–1.00; N = 46) with a sensitivity and a specificity of 100% and 77.5%, respectively (Figure 1(C)). The positive predictive value and the negative predictive value was 60% and 90%, respectively (Table 4).

## 4 DISCUSSION

The present, retrospective study demonstrated that the LDH value and DIC score were significant predictive factors of ARE during induction therapy in patients with neuroblastoma. The combination of the LDH value and DIC score was also found to be an outstanding predictive factor.

High serum LDH levels are associated with a large tumor burden.^15^ Moreover, a high serum LDH level is an important biomarker for diagnosing ARDS.^16^ Previous case reports described some pediatric patients with cancer, including neuroblastoma, in whom ARE developed in the context of tumor lysis.^15^, ^17^, ^22^ The findings of the present study suggested that ARE may be an aspect of cytokine release syndrome secondary to tumor lysis.

Lysed tumor cells release a variety of cytokines in addition to intracellular enzymes (e.g., LDH), which can induce severe hypoxia by eliciting a systemic inflammatory response syndrome, eventually leading to multiorgan failure.^17^, ^18^ The systemic proinflammatory cytokines stimulate the vascular endothelium^19^ and prime blood phagocytes.^20^ The activated phagocytes, which release proteolytic enzyme and toxic oxygen species, increase permeability in both alveolar epithelial cells and vascular tissue.^21^ Consequently, the cytokine release leads to severe hypoxia. In view of this pathophysiology, a high serum LDH level was considered as a promising predictive factor of ARE in neuroblastoma.

The DIC score was also predictive of ARE risk. Several, previous reports of the association of neuroblastoma with DIC^23^, ^24^ reported that DIC is also frequently associated with ARDS. Gando et al. reported DIC associated with endothelial injury had prognostic value for ARDS development.^25^ DIC reflects an inflammatory disorder of the microvasculature. The derangement of coagulation and fibrinolysis in DIC is mediated by several proinflammatory cytokines^26^ which, together with endothelial injury, can lead to severe hypoxia.

In the present study, 11 of the 75 neuroblastoma patients developed ARE; 3 of the 11 patients experienced ARDS, and all 11 received respiratory support in the PICU. Although there are currently no studies focusing on ARE because of their low incidence, our data suggested that ARE may be more common among patients with advanced neuroblastoma. If the possibility of ARE development were able to be predicted using our scoring system, countermeasures could be taken, such as reducing the intensity of the first chemotherapy regimen or introducing a prephase treatment to avoid rapid tumor lysis in the very early phase of induction chemotherapy.

Two types of ARE were identified during induction therapy in the neuroblastoma patients, including early onset pleural effusion and late onset ARDS or pericardial effusion. Early onset pleural effusion is thought to be caused by infiltration of the neuroblastoma,^27^ and late onset ARDS and pericardial effusion are thought to be caused by SRS secondary to TLS.^22^, ^28^ A cytokine profiling study is currently being conducted to investigate further the pathogenesis of ARDS and pericardial effusion in the context of tumor lysis.

Our study has some limitations. First, because it was retrospective, it may have included various biases, such as the sampling bias. Second, the small sample size might have led to an underestimation of the influence of various factors on univariate analysis while also precluding the use of multivariate analysis. Third, the DIC score was missing in 38.6% of the patients. Fourth, ARE was chosen as the outcome. Because many of the values required for arterial blood gas analysis were missing, oxygen saturation alone was used to assess the respiratory disorders. In view of these limitations, the reproducibility of our scoring system should be confirmed with a fairly large cohort in a nation‐wide, prospective clinical trial. If its reproducibility is confirmed, interventions, such as reduced‐intensity initial chemotherapy for patients with a high risk of ARE, should be tested prospectively.

## 5 CONCLUSION

The present study tested the hypothesis that the LDH value and DIC score could serve as predictive factors of ARE during induction therapy in patients with neuroblastoma. Identifying the predictive factors of ARE may enable us to prepare for it and to increase the chances of rescuing a patient with severe ARE.

## CONFLICT OF INTEREST

The authors have stated explicitly that there are no conflicts of interest in connection with this article.

## AUTHOR CONTRIBUTIONS

**Atsushi Makimoto:** Conceptualization; methodology; writing‐review & editing. **Nobuhiro Nishio:** Conceptualization; writing‐review & editing. **Yoshiyuki Takahashi:** Conceptualization; data curation; methodology; resources; writing‐review & editing. **Mitsuyoshi Urashima:** Conceptualization; formal analysis; investigation; methodology; writing‐review & editing. **Yuki Yuza:** Writing‐review & editing.

## ETHICAL STATEMENT

The Tokyo Metropolitan Children's Medical Center institutional review board approved this study. The ethical committee waived patient consent because of the retrospective and non‐interventional nature of the study (H30b‐258).

## Data Availability

The data that support the findings of this study are available from the corresponding author upon reasonable request.
